# Kill Four Birds With One Stone: Ablation of Atrial Fibrillation, Atrial Flutter, and Supraventricular Tachycardia Plus Left Atrial Appendage

**DOI:** 10.14740/jmc5352

**Published:** 2026-07-28

**Authors:** Zuo Feng Qin, You Wu, Yu Zhan, San Wu Wu, You En Zhang

**Affiliations:** a Institute of Clinical Medicine and Department of Cardiology, Renmin Hospital, Hubei University of Medicine, Shiyan, Hubei 442000, China; b Arteriosclerosis Cardiovascular Disease Clinical Medical Research Center of Hubei Province, Shiyan, Hubei 442000, China; c These authors contributed equally to this work.

**Keywords:** Atrial fibrillation, Catheter ablation, Left atrial appendage occlusion

## Abstract

Atrial fibrillation (AF) is the most common arrhythmia in clinical practice. Catheter ablation, particularly pulmonary vein isolation, reduces AF recurrence by 50% and is more effective than antiarrhythmic drugs. To better understand this condition, we present a case of a patient with persistent AF admitted to our department. The planned treatment strategy was a one-stop procedure combining catheter radiofrequency ablation with left atrial appendage occlusion (LAAO). Although AF, atrial flutter (AFL), and supraventricular tachycardia (SVT) exhibit distinct electrocardiographic characteristics, they often share overlapping triggers and common electrophysiological substrates within the atrial myocardium. AF is frequently initiated by ectopic triggers originating from the pulmonary veins, typical AFL is maintained by a macro-reentrant circuit around the cavotricuspid isthmus, and atrioventricular nodal reentrant tachycardia (AVNRT), the most common form of SVT, involves dual conduction pathways within the atrioventricular node. Transcatheter intervention provides an integrated therapeutic strategy that enables sequential targeting of these anatomically and electrophysiologically distinct substrates within a single electroanatomical mapping procedure, thereby offering a comprehensive curative approach while avoiding redundant interventions. However, the patient developed AFL and dual-pathway SVT during the procedure. Despite these challenges, radiofrequency ablation was performed successfully, followed by satisfactory LAAO.

## Introduction

Atrial fibrillation (AF) is the most common clinical arrhythmia, with increasing incidence and prevalence, affecting over 33 million people worldwide [1, 2]. Effective treatment of patients with AF includes the management of comorbidities and risk factors, with behavioral changes implemented to reduce the likelihood of AF episodes and to alleviate its burden. Previous studies have shown that for persistent and paroxysmal AF, catheter ablation is more effective than antiarrhythmic drugs. In particular, pulmonary vein isolation (PVI) reduces the recurrence of AF by 50% and significantly alleviates the burden of AF [3]. From a mechanistic perspective, AF and atrial flutter (AFL) are closely interrelated and may act as reciprocal arrhythmogenic substrates. Rapid, localized atrial activity in AF can organize into, or transition toward, the macro-reentrant circuit characteristic of AFL, most commonly involving the cavotricuspid isthmus (CTI). Conversely, the coexistence of supraventricular tachycardia (SVT), particularly atrioventricular nodal reentrant tachycardia (AVNRT), and AF is a well-recognized clinical phenomenon. Episodes of SVT with rapid atrial rates may promote acute electrical remodeling and increased atrial wall stress, thereby facilitating the initiation of AF in susceptible individuals. Catheter ablation remains a definitive therapeutic option for these arrhythmias, and the use of high-density electroanatomical mapping allows for precise, sequential identification and targeting of key substrates, including the pulmonary vein triggers, the CTI-dependent flutter circuit, and the slow pathway region of the atrioventricular node. To better understand this condition, here we present a patient with persistent AF admitted to our department, who was planned to undergo a one-stop treatment consisting of catheter radiofrequency ablation combined with left atrial appendage occlusion (LAAO). Despite the occurrence of AFL and dual-pathway SVT during the procedure, the planned catheter ablation and LAAO were completed successfully. To our knowledge, this is the first reported case describing the simultaneous management of persistent AF, typical AFL, AVNRT, and LAAO in a single integrated procedure. The patient was discharged with significantly improved condition.

## Case Report

### Investigations

A 72-year-old woman was admitted to our department due to palpitations and chest tightness lasting for over half a month. Medical history included persistent AF (lasting 14-month history), coronary artery disease, heart failure, hypertension, diabetes, and lacunar cerebral infarction. She had no history of smoking or alcohol consumption. Her pulse rate was 73 beats per minute, blood pressure was 144/80 mm Hg, and respiratory rate was 20 beats per minute. The patient was conscious and well-oriented during the physical examination. No bleeding or jaundice was observed on the skin or mucous membranes, and there was no jugular venous distention. The thyroid gland was not enlarged. Coarse breath sounds were present in both lungs, with moist rales heard at the lung bases. The heart rate was 105 bpm with an irregular rhythm. The abdomen was soft, with no tenderness or rebound tenderness. The liver and spleen were not palpable below the rib cage, hepatojugular reflux sign was negative, and there was no edema in the lower limbs.

### Diagnosis

Blood routine, liver and kidney function tests, coagulation function, thyroid function, and troponin tests showed no significant abnormalities. Triglyceride level was 2.39 mmol/L, low-density lipoprotein–cholesterol level was 1.95 mmol/L, NT-proBNP level was 1,628 pg/mL, and glycated hemoglobin was 6.60%. Electrocardiogram (ECG) indicated AF. The LAA depth and orifice diameter measured during preoperative transesophageal echocardiography (TEE) were 47 and 19–23 mm, respectively. Additionally, TEE showed slow blood flow in the left atrium with a slight smoke-like echo and blood stasis in the LAA with a significant smoke-like echo, with no obvious solid masses observed. Stroke risk score (CHA_2_DS_2_-VASc) and bleeding risk score (HAS-BLED) were 4 and 3, respectively. A one-stop surgical procedure consisting of radiofrequency ablation for AF and LAA occlusion was proposed.

### Surgical procedure

**Figure 1:**
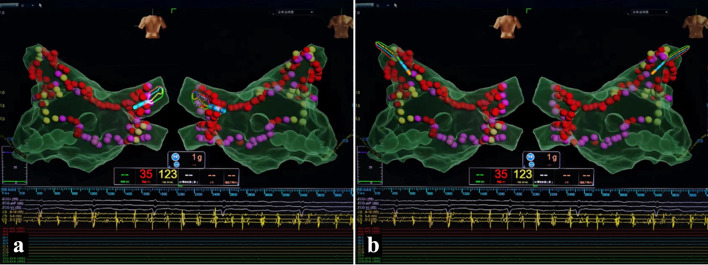
Pulmonary vein isolation. (a) Right pulmonary vein isolation. (b) Left pulmonary vein isolation.

**Figure 2:**
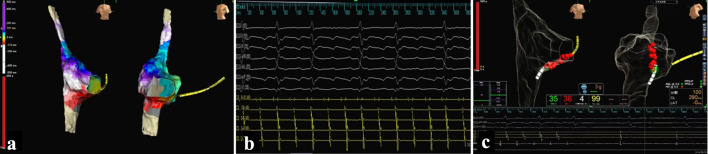
Electrophysiological examination and ablation of atrial flutter. (a) Early activation on CS9-0, indicating right atrial origin of flutter. (b) Mapping of the right atrium using an HD-Grid mapping catheter revealed counterclockwise cavo-tricuspid isthmus-dependent atrial flutter. (c) Pressure-sensing saline-irrigated catheter was used as the ablation catheter to create a line across the tricuspid isthmus. The power was set at 35 W, the temperature at 43 °C, and the saline irrigation rate at 17 mL/min. The sinus rhythm was restored during the ablation procedure.

**Figure 3:**
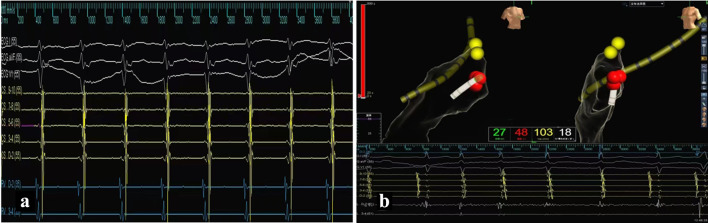
Electrophysiological examination and ablation of supraventricular tachycardia. (a) Dual-pathway supraventricular tachycardia (SVT) was induced. (b) Target points for ablation were identified in the slow-pathway region. Using a 50 W, 55 °C temperature-controlled mode, effective ablation was indicated by the appearance of slow junctional rhythm, consolidated for 120 s, and confirmed by the lack of recurrence after the procedure.

**Figure 4:**
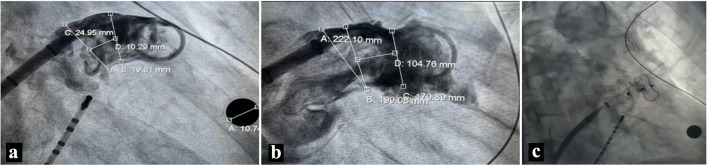
Left atrial appendage (LAA) occlusion. (a) The asymmetry of the LAA’s upper and lower edges was noted, with a longer upper ridge, measuring a fixation zone of 17–19 mm, and an LAA orifice of 20 mm. (b) With a clear landing zone, a 20–26 mm LACBES occluder was selected for occlusion. (c) The successful deployment of the occluder, which indicated it can be released according to PAST principles.

The patient had persistent AF and developed more AF episodes during the surgical procedure. In accordance with the 2024 ESC Guidelines for the Management of Atrial Fibrillation and the 2023 HRS/APHRS/LAHRS Expert Consensus Statement, which suggest that substrate modification beyond PVI may be considered in patients with persistent AF and signs of advanced structural or electrical remodeling, a posterior wall isolation (BOX lesion) strategy was pursued to eliminate potential non-pulmonary vein drivers and rotors in the fibrotic posterior wall. To achieve PVI, a bilateral pulmonary vein electrical isolation combined with roof line and bottom line (BOX) ablation was initially performed (Fig. 1a, b). However, the patient experienced AFL, and coronal sinus (CS) catheter recorded early activation on CS9-0, indicating right atrial origin (Fig. 2a). Electrophysiological mapping revealed a flutter cycle length (FCL) of 240 ms. Using an HD-Grid high-density mapping catheter, mapping of the right atrium was performed, revealing counterclockwise cavo-tricuspid isthmus-dependent AFL (Fig. 2b). Entrainment mapping from the CTI demonstrated a post-pacing interval (PPI) minus FCL of 15 ms, confirming CTI-dependency. Ablation was performed along the tricuspid isthmus with a pressure saline ablation catheter at 35 W and 43 °C, with a saline flow of 17 mL/min, leading to conversion to sinus rhythm (Fig. 2c). Following this, dual-pathway SVT was induced (Fig. 3a). Evidenced by a clear A-H jump (an increase in the AH interval by 65 ms with a 10-ms decrement in S2), which immediately induced a narrow QRS tachycardia with a tachycardia cycle length (TCL) of 360 ms. The V-A interval measured during tachycardia was short (< 70 ms), with the earliest atrial activation recorded at the His bundle region. Ventricular entrainment pacing during tachycardia demonstrated an “A-V” response upon termination of pacing, with a PPI-TCL > 110 ms, effectively ruling out orthodromic atrioventricular reentrant tachycardia (AVRT) and atrial tachycardia, thereby securing the definitive diagnosis of typical slow-fast AVNRT. Target points for ablation were identified in the slow-pathway region. Using a 50 W, 55 °C temperature-controlled mode, effective ablation was indicated by the appearance of a slow junctional rhythm, consolidated for 120 s, and confirmed by the lack of recurrence after the procedure (Fig. 3b). Next, LAAO was performed. Although digital subtraction angiography (DSA) indicated a high anterior interatrial septal puncture site with poor axial alignment, the sufficient LAA depth made the occlusion feasible in this case. Hepatic position angiography (RAO30, CAUD20) showed a windsock-shaped LAA with asymmetrical upper and lower edges, with a longer upper ridge. Preoperative TEE demonstrated a windsock-shaped left atrial appendage with a clear landing zone, the fixation zone was 17–19 mm, and the diameter of the LAA orifice was 20 mm (Fig. 4a). With a clear landing zone, a 20–26 mm LACBES^®^ occluder was selected for occlusion (Fig. 4b). The anchoring cylinder was deployed in the usual manner, forming a classic “tire-like” shape, adhering well to both the upper and lower LAA walls. The device position, anchoring stability, size adaptation, and sealing effect were systematically evaluated according to the PASS/PAST criteria, all of which were satisfactorily fulfilled prior to release.

### Follow-up and outcomes

The sealing disc was deployed, and a tug test showed no movement. Immediate angiography confirmed effective occlusion with no residual shunt. No device-related thrombus or residual leakage was observed intraoperatively. The occluder was successfully released after confirming the stability per PAST principles, achieving satisfactory occlusion (Fig. 4c). Postoperative TEE follow-up was not performed in this single case, which is acknowledged as a limitation of this report.

## Discussion

The incidence and prevalence of AF are increasing, making it one of the greatest global epidemics and public health challenges of the future that significantly reduces the patients’ quality of life. According to the Optum database of US commercial insurance, patients with AF have higher rates of hospital admissions, cardiovascular-related emergency visits, and healthcare costs than those without AF [4]. Epidemiological data indicate that the overall lifetime risk of AF is about 30–40% among the white population and around 20% among Chinese [5]. Previous studies have shown that paroxysmal AF combined with AFL is common in clinical practice, with a complex pathogenesis. In terms of treatment, catheter ablation has been recognized as the preferred treatment option for curing paroxysmal AF combined with AFL. The safety and efficacy of circumferential PVI have been validated. However, its effectiveness remains insufficient for persistent AF. According to Brembilla-Perrot et al [3], catheter ablation is more effective than antiarrhythmic drugs for both persistent and paroxysmal AF. Treatment including PVI reduces the occurrence of recurrent AF by 50% and is more effective than drug therapy in reducing the burden of AF. In most cases, combined paroxysmal AF and AFL originate from the pulmonary veins, and circumferential PVI can treat most patients suffering from such conditions. A study by Hsieh et al [6] involving 54 patients with AF combined with AFL showed that 85% of ectopic beats originated from the pulmonary veins. Ablation of these trigger foci eliminated the transition from AFL to AF. AF combined with AFL may also have non-pulmonary vein trigger foci, so simple PVI is not ideal for some cases of AF combined with AFL. Current expert consensus suggests that ablation should target not only AF-triggering foci, but also the substrate that maintains fibrillation outside the pulmonary veins, a strategy known as substrate modification [7]. In this case, the patient’s AF was treated using the strategy of bilateral pulmonary vein electrical isolation combined with roof line and bottom line (BOX) ablation. After terminating AF, the patient developed counterclockwise cavo-tricuspid isthmus-dependent AFL, which converted to sinus rhythm during the ablation process.

Compared with conventional one-stop procedures that typically involve AF ablation combined with LAAO, this case demonstrates a broader electrophysiological strategy. In addition to AF substrate modification, intraoperative arrhythmia induction enabled the identification and elimination of CTI-dependent AFL and AVNRT, which are often not diagnosed in routine procedures.

Research on the correlation between SVT and AF was mostly conducted over a decade ago with a focus on analyzing the occurrence of AF in patients who underwent surgical treatment of SVT. Some studies suggest that the long-term incidence of AF in SVT patients is higher than that in the general population. However, research on the occurrence of SVT during AF surgical procedures is sparse. Atrial remodeling induced by long-standing AF may promote heterogeneous conduction properties within the atrioventricular node region, facilitating dual-pathway physiology and increasing susceptibility to AVNRT. Conversely, rapid atrial activation during AVNRT episodes may further exacerbate atrial electrical instability, potentially contributing to AF initiation and maintenance, indicating a possible bidirectional arrhythmogenic relationship. A considerable proportion of candidates for AF ablation are inducible for an SVT. SVT ablation shows a preventive effect on AF recurrences [8]. In this patient, radiofrequency ablation terminated AF and AFL, and subsequent electrical stimulation induced dual-pathway SVT. Ablation targeting the slow-pathway region was successful. In non-valvular AF patients, 90% of thrombi are formed in the LAA. Multiple clinical trials have confirmed that transcatheter LAAO effectively prevents thrombus formation in the LAA during AF, thereby reducing the risk of long-term disability or death from thromboembolic events [9]. The elderly female patient in this report had high stroke and bleeding risk scores (CHA_2_DS_2_-VASc = 4, HAS-BLED = 3), and the strategy was to perform LAAO after radiofrequency ablation.

To the best of our knowledge, this is the first case in which AF, CTI-dependent AFL, inducible AVNRT, and LAAO were successfully managed within a single procedural strategy. This case suggests that systematic intraoperative arrhythmia induction combined with high-density mapping may help uncover latent arrhythmogenic substrates in selected patients with persistent AF. Such a comprehensive strategy may reduce repeat procedures and improve long-term rhythm outcomes, although further studies are required to validate its broader applicability.

### Limitation of follow-up data

This case has an important limitation regarding the lack of long-term structured follow-up. The patient was unable to return for scheduled outpatient evaluations (including Holter monitoring and TEE) due to traveling with family after discharge. Therefore, systematic assessment of long-term rhythm outcome, device status, and anticoagulation strategy could not be obtained. Instead, follow-up was performed via telephone communication. At the latest contact, the patient remained in sinus rhythm and reported no recurrence of AF, AFL or SVT, and no symptoms of heart failure, thromboembolic events, or bleeding complications. However, the absence of imaging-based and device-related follow-up data limits definitive evaluation of long-term procedural efficacy and safety of this one-stop strategy.

With the continuous advancement and expanding indications of radiofrequency ablation technology, the ablation of complex atrial tachycardia has become a frequent challenge for electrophysiologists. We learned from this case that a solid understanding in electrophysiology is crucial for diagnosing and treating arrhythmias. Attention to detail helps in decision-making such as positioning of catheter, recognizing abnormal electrode potentials, and substrate mapping. High-density mapping aids in the identification and ablation of atrial arrhythmias, improving efficiency and accuracy, and making it easier to detect abnormal electrode potentials.

### Learning points

This case highlights that the importance of solid basic electrophysiological knowledge and judgment of intraoperative details are the cornerstones of arrhythmia diagnosis and treatment, which is likely that a second operation will be avoided in the patient.

## Data Availability

The data that support the findings of this case report are available from the corresponding author upon reasonable request.

## Abbreviations

AF: atrial fibrillation
LAA: left atrial appendage
PVI: pulmonary vein isolation
SVT: supraventricular tachycardia
TEE: transesophageal echocardiography
